# When Do We Start Caring About Insect Welfare?

**DOI:** 10.1007/s13744-022-01023-z

**Published:** 2023-01-19

**Authors:** Tina Klobučar, David N. Fisher

**Affiliations:** School of Biological Sciences, University of Aberdeen, King’s College, Aberdeen, UK

**Keywords:** Entomophagy, Insect farming, Insect sentience, Insect welfare, Minilivestock, Pain

## Abstract

The world is facing an incoming global protein shortage due to existing malnutrition and further rapid increases in population size. It will however be difficult to greatly expand traditional methods of protein production such as cattle, chicken and pig farming, due to space limitations and environmental costs such as deforestation. As a result, alternative sources of protein that require less space and fewer resources, such as insects and other invertebrates, are being sought. The Neotropics are a key area of focus given the widespread prevalence of entomophagy and developing animal welfare regulations. Unlike vertebrate livestock however, insect “minilivestock” are typically not protected by existing animal welfare regulations. This is despite the fact that the evidence is mounting that insects possess “personalities”, may experience affective states analogous to emotions and feel something like pain. In this forum article, we highlight this discrepancy, outline some of the emerging research on the topic and identify areas for future research. There are various empirical and ethical questions that must be addressed urgently while insect farming is ramped up around the globe. Finally, we describe the benefits and also potential costs of regulation for insect welfare.

## Bugs to plug the protein gap

Humanity is facing an imminent global protein shortage. The combination of growing populations and the additional need to provide nutrition for the one billion people currently undernourished means that meat production needs to double from 1991 levels by 2050 (Steinfeld et al. 2006). However, 70% of current arable land is already used to grow feed for meat production, and this would have to increase by 66% if all nations consumed meat at levels equivalent to those in the Western world (Steinfeld et al. 2006). Furthermore, livestock contributes 18% of greenhouse emissions and plays a huge role in land degradation, with pasture for livestock now using 70% of previous forested land in the Amazon (Steinfeld et al. 2006). Increasing the numbers of traditional livestock to meet the incoming protein shortage is therefore untenable without drastic environmental destruction.

**Fig. 1:**
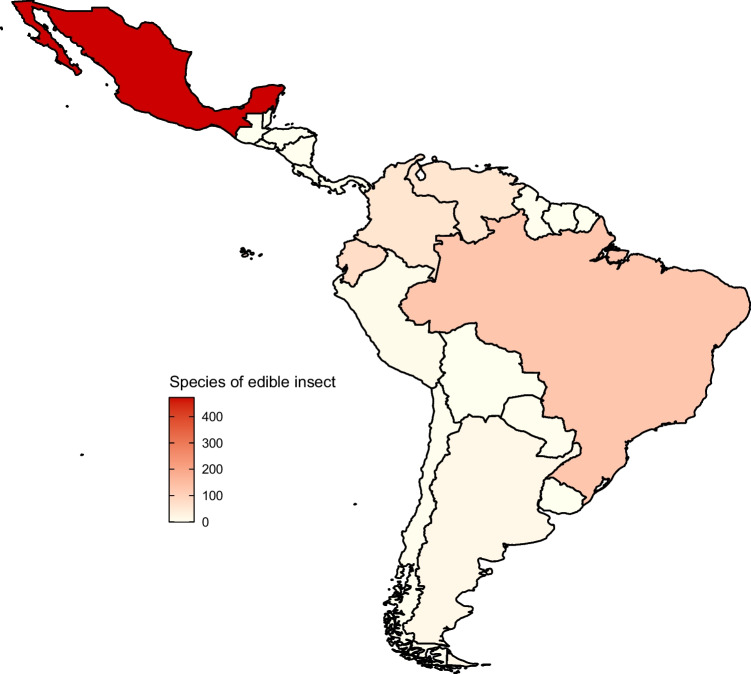
A map of Mexico, Central and South America, showing the number of different insect species eaten in each country. Darker countries indicates a higher number of species eaten. Data from https://www.wur.nl/en/research-results/chair-groups/plant-sciences/laboratory-of-entomology/edible-insects/worldwide-species-list.htm, compiled by Mr. Yde Jongema, last updated 01/04/2017. Figure produced in R (ver. 4.2.1)

To meet the global demand, people are starting to look to insects and other invertebrates as farmable sources of protein for both humans and vertebrate livestock. Insects of 1900 different species are already eaten in 80% of nations (Lilholt 2015), and so entomophagy is often suggested as a readily available solution. In the Americas, 39% of the population is entomophagous, making it the leading continent in insect consumption (Morimoto 2020), while Latin America has the second largest market for edible insects in the world (Bermúdez-Serrano 2020; see Fig. 1). Peoples from 3071 different ethnic groups across Argentina, Colombia, Mexico, Venezuela, Peru, Brazil and Ecuador consume insect species (Ramos-Elorduy 2009; Costa-Neto 2015, 2016; Loiacono et al. 2016). For example, in Mexico, a total of 29 ethnic groups such as the Tlapaneco, Maya and Yutoazteca consume Lepidoptera spp. (Ramos-Elorduy et al. 2011; Gómez et al. 2016; see also: Hurd et al. 2019), while the diet of the Yukpa-Yuko ethnic group in Venezuela and Colombia includes 22 insect genera (Ruddle 1973; Paoletti et al. 2000). In addition, many of the tribes in the Amazon Basin are entomophagous (Paoletti et al. 2000). Therefore, there is extensive precedent for using insects as a source of protein. Furthermore, insects convert a higher proportion of feed to protein than mammalian and avian livestock (Nakagaki and Defoliart 1991) and have lower water requirements (van Huis 2013). This high efficiency implies they have great potential to meet protein shortages for low financial and environmental costs (van Huis 2013).

While great challenges remain in converting small-scale farming and laboratory experiments on insects to cost-effective industrial-scale protein production, not to mention convincing Westerners to consume the results (van Huis 2013), the possibility of farming insects throws into spotlight the welfare of these “minilivestock” (Paoletti 2005; van Huis 2021). The specifics vary from country to country, but mammalian and avian livestock have their welfare during rearing, transport and slaughter protected in various ways, with requirements possibly including a limit on the number of animals per square meter (stocking density), access to a suitable environment, diet, the opportunity to exhibit normal behaviour patterns and to be protected from suffering, injury and disease. While variation in practices and the duration they have been implemented exist, in Latin America, more stringent animal welfare regulations are emerging, such as legislation changes and training in animal handling practices (Gallo et al. 2010; da Costa et al. 2012; Souza et al. 2019). This is due to the more restrictive regulations of export destination markets such as Europe, to increase profits by reducing meat loss (both economic concerns), and domestic consumer markets that are increasingly animal welfare conscious (a social concern; da Costa et al. 2012; Souza et al. 2019). Despite these advances being made for vertebrate welfare, these protections would be totally absent for farmed insects, as regulations typically name the specific livestock or refer to vertebrates in general (see the “invertebrate dogma”—Mikhalevich & Powell 2020). This lack of protection makes it possible to keep non-vertebrates in any conditions and do anything to them, including eating them alive (see: https://en.wikipedia.org/wiki/Eating_live_seafood). Having said that, according to van Huis (2021), invertebrates in the Netherlands are protected under the Animals Act once they start being farmed for food. This implies clear welfare requirements. Whether these regulations exist in other countries, and to what extent they are adhered to, needs to be verified.

## Tiny empathy for tiny minds?

The lack of welfare protection is potentially problematic as our knowledge of the world as experienced by insects grows (Drinkwater et al. 2019). Individual insects are known to have consistent differences in behaviour (known as “personality”; Dall et al. 2004), such as varying in their activity levels (Fisher et al. 2015), their degree of exploration and risk-taking behaviour (Niemelä et al. 2012), and in their sociability (Walton and Toth 2016). Insects are in fact often used as model species for studying why all animals may display personalities (Kralj-Fišer & Schuett 2014; Mather & Carere 2019).

Furthermore, insects may show states or levels of motivation similar to emotions (Perry and Baciadonna 2017). While most research on the mental world of insects focuses on their cognitive rather than their emotional capabilities (Lambert et al. 2021), some work has indicated insects possess the latter. For example, Bateson et al. (2011) conditioned honeybees (*Apis mellifera carnica*) to two odours, which either elicited a positive response—opening of the mouthparts, or a negative response—keeping the mouthparts closed. Half of the experimental bees was then shaken for 1 min, which mimicked the natural state of a beehive colony under attack by predators, leading to a negative affective state. Five minutes after being shaken, the bees were exposed to five odours, the two used previously during conditioning and three new ambiguous odours that were a combination of the two. The bees that were shaken after conditioning were more likely to keep their mouthparts closed when exposed to an ambiguous odour, indicating the negative stimuli had caused them to be in a negative affective state (Bateson et al. 2011).

Finally, invertebrates have been recorded to show some behaviours consistent with feeling pain, such as showing attention to the site of a wound and learning in response to the use of electric shocks as a method of reinforcement (Barr et al. 2008). For example, prawn (*Palaemon elegans*) exposed to noxious stimuli, such as acetic acid and pinching, show increased grooming of the site of the wound and rubbing of the affected antennae on the experimental tank wall (Barr et al. 2008). Similarly, honeybees (*A. mellifera*) can be taught to associate an odour with electric shocks, resulting in the extension of their stinger in response solely to the odour, demonstrating their learning abilities in response to pain (Vergoz et al. 2007). Physiological responses such as neuron hypersensitivity after injury help with healing and enhance natural escape responses (Crook et al. 2014; Gu et al. 2022). *Drosophila melanogaster* shows neuron hypersensitivity and the dysregulation of the system causes prolonged hypersensitivity, analogous to chronic pain (Gu et al. 2022; McParland et al. 2021; Khuong et al. 2019). Furthermore, pathways underpinning changes in sensitivity, such as Trp channels, are conserved among flies, mice and humans (Neely et al. 2010). This tells us that we can expect invertebrates to feel pain in some form, and therefore, their welfare from that perspective, as well as economic and social perspectives, needs to be accounted for.

While the insect versions of personality, emotion and pain may not match the human experience or be as close to it as of other mammals and birds, the fact that insects are not simply robots and are sentient in their own right clearly advocates for some consideration of insect welfare. However, scientific uncertainty remains about the extent of insects’ cognitive complexity (Lambert et al. 2021). Their small brains may limit the sophistication of their sentience, which when combined with the traditional view of them being “lower on the scale of nature” than vertebrates, may have inhibited exploration of their mental capacities. Furthermore, insects are detested by a large proportion of the human population (Lorenz et al. 2014), triggering feelings of fear and disgust (Fukano and Soga 2021), although there is variation among species in how much disgust they elicit (Jose 2019). This is due to their unfamiliar appearance, smaller stature and morphological and behavioural differences (Prokop et al. 2011). Finally, despite being vital in essentially all ecosystems, invertebrates are not consistently acknowledged by the public as integral parts of the ecosystem as vertebrates are (Leandro and Jay-Robert 2019). In addition, there are fewer conservation efforts focused on invertebrates (Clark and May 2002). We therefore need to make a concerted effort to conduct research on insect sentience, and convince others to do so, in order to raise awareness of how these animals experience the world and that their welfare should be of concern.

## Looking out for minilivestock

A lack of knowledge, empathy and economic incentive are likely to limit the development of research programs designed to fully explore how the methods we use to rear, house, experiment on and kill insects impacts their welfare (Cammaerts 2020). This will need to change as the number and diversity of insects farmed for food grows. For example, testing which euthanasia methods are most humane is of top priority, as very large numbers of individuals will need to be killed to meet overall protein requirements. Euthanasia methods include freezing, boiling and shredding. These different approaches can be combined, e.g. insects can be cooled or frozen before shredding, or anesthetised with carbon dioxide before boiling (Bear 2019; Zhen et al. 2020). Luckily, methods to maximise production may often align with better welfare. For example, Adámková et al. (2017) showed that euthanasia by boiling (as opposed to freezing) and nutritional deprivation both negatively affected nutritional value and welfare in mealworms (*Tenebrio molitor*). More research like that of Adámková et al. (2017) is required to test how these different methods impact on welfare and economics. Furthermore, a goal that unifies the study of productivity and welfare is developing methods to ensure insects reared in captivity are free from disease, as this can both cause pain and suffering and dramatically impact productivity.

Alongside these methodological considerations, important ethical considerations also need to be considered. For example, although both a chicken and a cricket are a single, autonomous organism, that does not necessarily mean we value their lives equally, nor should we. Is it therefore reasonable to kill 200 insects to obtain 200 g of protein, if it means sparing a single chicken (Knutsson 2016; Scherer et al. 2018)? If the answer is yes, how many more insects would it take before you consider it an unreasonable substitution? This is a challenging question where a clear answer may remain elusive but consideration at least of the issue is vital.

For many of our outstanding questions, we lack the knowledge to be able to write guidelines. In the meantime, it is therefore an important debate whether we should use the “precautionary principle” and assume insects can experience pain unless proven otherwise (adopting: “absence of evidence is not evidence of absence”). However, that may be too restrictive, and instead, we maybe should adopt the “appropriate burden of proof of sentience”, where evidence from experiments that meet normal scientific standards needs to be obtained in at least one species of a given order before assuming cognitive processes such as sentience or the feeling of pain in that order (Birch 2017). Resolving this question satisfactorily will fundamentally change the pace and scope of the necessary research to be carried out.

A consideration of insect welfare does not rule out using them for food production; in the same way, it should not rule out their use in scientific research (reviewed in Drinkwater et al. 2019). Instead, it just requires us to use a consistent set of ethical standards when farming invertebrates as we do when farming vertebrates. This does not necessarily mean the same standards. Given the numbers of individuals likely to feature in insect food production systems, their small size and different physiology, applying the welfare standards currently in place for vertebrate livestock to insects would simply be intractable and cripplingly expensive. This would halt the insect food production industry before it gets off the ground, something that could be enormously costly for the planet as a whole. Insects may also genuinely experience equivalent stimuli differently to vertebrates, which would justify different treatment. Additionally, there are various aspects of life histories in invertebrates that might not be present in vertebrates, such as metamorphosis (Kralj-Fišer and Schuett 2014), and some argue that the fact that we have no analogous experience of this kind means that the farming of insects should be precautionary (van Huis 2021). Scientific and medical knowledge can greatly benefit from being able to conduct experimental work on insects and other invertebrates that would be difficult or impossible if they were afforded the same protections as vertebrates (Freelance 2019). A core tenement in medical and scientific research is to use less cognitively sophisticated organisms if possible, and there is no reason for this to change. We simply must appreciate the cognitive sophistication of insects, not simply view them as objects for gastronomic or research purposes (Horvath et al. 2013), and therefore treat them appropriately when considering their increased use in the food industry (Santaoja and Niva 2019).

Instead, new regulations are required, perhaps based on the “five freedoms” of freedom from hunger and thirst, from discomfort, from pain injury or disease, from fear and distress and the freedom to express normal behaviour (Brambell 1965; Farm Animal Welfare 1979). This might mean regulations for unrestricted or regular access to feed and hydration, requirements for shelter in all housing, maximum stocking densities and limited mixing of different ages and sizes if cannibalism is a risk. Given the diversity of invertebrate forms and behaviours, it is conceivable that different final criteria are established for different groups, especially for species that differ in the life stage that is harvested. Ultimately, we return to the point that no regulations at all seems difficult to justify given all we know now about the cognitive sophistication of insects, but we emphasise that vertebrate-level regulations are most likely unnecessary and unworkable (Baracchi and Baciadonna 2020).

Moving forward, we need to make advances on several fronts. First, we need clarity on whether any existing animal welfare regulations include insects, if these regulations are adhered to, and what impact that has had. We then need to better understand how the methods we use to rear, house and kill insects impact both yields and welfare, and if these are complementary or contrasting goals. Alongside this, we need discussions involving scientists and members of the public on several ethical questions, such the validity of substituting some number of insect lives for one vertebrate life, and how much lower it is reasonable to set the bar for invertebrate welfare than vertebrate welfare, if we set it lower at all. Finally, we need to increase the awareness of other scientists, policymakers and the public of the extent of our knowledge of the diversity and scope of the lived experiences of insects, so that all discussions moving forward take place on a sound scientific footing. Overall, this work will require collaboration between researchers in a range of disciplines to help determine how insects experience the world and therefore what degree of protection their welfare should receive.
